# *Ekueitermes alerosinus*, a new genus and species of soldierless termite from Colombia (Isoptera, Termitidae, Apicotermitinae)

**DOI:** 10.3897/zookeys.1292.195853

**Published:** 2026-09-18

**Authors:** Ervin H. Duran-Bautista, Fernando Celis-Daza, Nasly S. Poveda Quezada, Fausto A. Ortiz-Morea, Rudolf H. Scheffrahn, Daniel Castro

**Affiliations:** 1 Facultad de Ingeniería, Ingeniería Agroecológica, Universidad de la Amazonia, Calle 17 Diagonal 17 Carrera 3F, Florencia, Caquetá, Colombia Facultad de Ingeniería, Ingeniería Agroecológica, Universidad de la Amazonia Florencia Colombia; 2 Laboratorio de Ecología del Suelo, Universidad de la Amazonia, Calle 17 Diagonal 17 Carrera 3F, Florencia, Caquetá, Colombia Laboratorio de Ecología del Suelo, Universidad de la Amazonia Florencia Colombia; 3 Grupo de Investigación en Agroecosistemas y Conservación en Bosques Amazónicos-GAIA, Florencia, Caquetá, Colombia Grupo de Investigación en Agroecosistemas y Conservación en Bosques Amazónicos-GAIA Florencia Colombia; 4 University of Florida, Fort Lauderdale Research & Education Center, 3205 College Avenue, Davie, Florida, USA University of Florida, Fort Lauderdale Research & Education Center Davie United States of America; 5 Facultad de Ciencias Agrarias, Universidad Nacional de Colombia, Bogotá, D.C., Colombia Facultad de Ciencias Agrarias, Universidad Nacional de Colombia Bogotá Colombia

**Keywords:** Apicotermitinae, Colombian Amazon, enteric valve, molecular phylogeny, Neotropics, soil-feeder

## Abstract

A new genus and species of soldierless termite, *Ekueitermes alerosinus***gen. et sp. nov**., is described from Colombia based on morphological and molecular evidence. The diagnostic characters for the new taxon are a mixed segment with a short, non-inflated mesenteric tongue, a very short and tubular P1, a well-differentiated tubular enteric valve seating, and an enteric valve armature with six cushions armed with 7–17 sclerotised spines arranged in a palmate shape and polygonal scales. Furthermore, Bayesian inference and maximum-likelihood phylogenetic analysis using concatenated COI and 16S genes, including other Neotropical Apicotermitinae genera described to date, supports the new genus as a distinct terminal and the sister group to *Disjunctitermes insularis*.

## Introduction

Soil-feeding termites play a crucial role in the recycling of organic matter and nutrient dynamics, which directly impact the functioning of tropical ecosystems (Eggleton 2011; Duran-Bautista et al. 2020). Among them, the subfamily Apicotermitinae stands out for being composed almost entirely of soil-consuming species (Bourguignon et al. 2016a). However, this group exhibits interesting ecological plasticity; for instance, genera such as *Ruptitermes* Mathews, 1977 and *Aparatermes* Fontes, 1986 have shifted towards specialised foraging behaviours, feeding primarily on leaf litter and organic debris at the soil surface rather than strictly processed humus (Acioli and Constantino 2015; Pinzón et al. 2019). Their diversity is especially high in the Neotropics, where they can represent up to half of the termite species in some assemblages (Carrijo et al. 2009; Bourguignon et al. 2011; Palin et al. 2011; da Silva et al. 2019; Castro et al. 2021; Ferreira et al. 2023).

The development of apicotermitine taxonomy has been gradual. The subfamily was first proposed by Grassé and Noirot (1955) and later defined by Sands (1972) in the most extensive taxonomic work on the group in Africa. In the Neotropics, knowledge remained limited for decades; before 2009, only five genera had been described (Fontes 1986; Bourguignon et al. 2010). However, recent advances in morphology and molecular phylogeny have led to a substantial increase in recognised taxa, with 67 species belonging to 25 genera currently recorded in the Neotropics (Constantino 2025). Despite this progress, various studies suggest that many lineages remain undescribed, reflecting the large and still hidden within the termite fauna of the region (Bourguignon et al. 2013).

Parallel to this taxonomic growth, the methodology for delimiting and describing genera and species has undergone a significant transformation. While classical descriptions traditionally relied on external imago morphology (e.g. Silvestri 1901; Emerson 1925), later descriptions included internal anatomy of the worker caste such as the enteric valve armature and gut coiling (Sands 1972). Recent studies have shifted toward an integrative approach. Descriptions of new genera in this subfamily now increasingly incorporate molecular data, predominantly mitochondrial markers (Bourguignon et al. 2016b; Castro et al. 2020; Carrijo et al. 2023). While many recent taxonomic works rely on a single molecular marker (most commonly COI), the use of multiple genes provides a more robust framework for establishing phylogenetic relationships. These markers have proven essential for delimiting lineages where morphological convergence, or the inherent absence of a soldier caste, complicates traditional identification.

Following this integrative trend, we herein describe the worker of *Ekueitermes alerosinus* gen. et sp. nov., providing both a detailed morphological characterisation and complementary molecular evidence from COI and 16S rRNA sequences to define its phylogenetic placement within the Neotropical Apicotermitinae.

## Methods

The material examined in this study was deposited in the Central Taxonomic Collection in liquid at the “Laboratorio de Entomología de la Universidad de la Amazonia” (**LEUA**), in Florencia, Caquetá, and the University of Florida Termite Collection (**UFTC**; Scheffrahn 2019), in Davie, Florida. All specimens were preserved in 80–85% ethanol.

### Morphological analysis

Morphological measurements and imaging procedures were conducted according to the specific equipment available at each facility. At LEUA, measurements were performed using an Olympus SZ61 stereoscope equipped with a U-OCMC10/100X ocular micrometer and an Olympus 110AL2X-2 auxiliary lens, while images were captured with a Leica M205A stereomicroscope and a Leica DFC450 camera, subsequently processed using the Leica Application Suite software. Procedures at UFTC involved positioning preserved workers in a transparent Petri dish filled with Purell® hand sanitizer (70% EtOH) for stability during imaging. Body and head sections were then photographed as multi-layer montages using a Leica M205C stereomicroscope and a Leica DFC 425 module. For the detailed analysis of the enteric valve armature (EVA), the valve was mounted on a slide using PVA mounting medium (Bioquip Products, Inc.) and photographed with a Leica CTR 5500 compound microscope under bright-field lighting, utilising the same montage software. The distribution map was elaborated using ArcGIS desktop v. 10.8 (ESRI, Redlands, CA).

A morphological analysis of the EVA and digestive system in the worker caste was carried out. The nomenclature used to describe both the digestive tract and the mandibles followed the terms defined by Noirot (2001), Sands (1972), and Deligne (1999). We used the term “molar process” proposed by Constantini et al. (2020). Several morphometric characters were recorded following the nomenclature of Roonwal (1970). Workers including: length from head to base of mandibles (5); maximum head width (17); length of the protibia (85); width of the protibia (86); ratio between width and length of the protibia (index 53).

### DNA extraction

DNA was extracted from three independent pools, each consisting of a pool of five termites. Genomic quality was verified via spectrophotometry (Nanodrop One, Thermo Fisher) and 1.2% agarose gel electrophoresis using Nancy-520 (Sigma-Aldrich). Two mitochondrial regions were amplified: COI using universal primers LCO1490 and HCO2198 (Folmer et al. 1994), and 16S rRNA using LR-N (Simon et al. 1994) and LR-J (Kambhampati and Smith 1995). Purified PCR products were sequenced via the Sanger method (ABI Prism 3730; PE Applied Biosystems, Foster City, CA, USA). Reactions were prepared with JumpStart™ Taq ReadyMix™ (Sigma-Aldrich), 1 μL of each primer (10 μM), and approximately 15 ng of genomic DNA in a 50 μL final volume. Sequencing was performed in both directions using the Sanger method on an ABI Prism 3730 automatic sequencer following standard protocols. Chromatograms were inspected in FinchTV v. 1.4.0 (Geospiza Inc. 2004, Seattle, WA, USA), trimming low-quality ends.

### Phylogenetic relationships

Phylogenetic analyses were based on a total of 34 terminals, including the sequences generated in this study (COI and 16S) and 32 published records of Apicotermitinae and outgroups retrieved from GenBank (mostly derived from complete mitochondrial genomes; Suppl. material 1). *Sphaerotermes sphaerothorax* was used as the outgroup. Sequences were aligned per marker using the MUSCLE algorithm in MEGA v. 12 (Kumar et al. 2024) and concatenated in AMAS (Borowiec 2016) into a final matrix of 941 bp. Haplotypes were identified with DnaSP v. 5.10 (Rozas 2009). Genetic distances were estimated in the same program under the Kimura 2-parameter (K2P) model, applying the pairwise deletion option for ambiguous positions (Suppl. material 6).

Phylogenetic relationships were inferred under both Bayesian inference (BI) and maximum-likelihood (ML) criteria. BI was performed in BEAST v. 2.5 (Bouckaert et al. 2019) using a Yule speciation prior and the TN93 substitution model with estimated base frequencies for both markers. The MCMC analysis was run for 10 million generations, sampled every 1,000 generations. Convergence and stationarity were verified in Tracer v. 1.7 (Rambaut et al. 2018), with all effective sample sizes exceeding 200. After discarding the first 10% of samples as burn-in, a maximum clade credibility tree was generated in TreeAnnotator and visualised in FigTree v. 1.4.4. ML analysis was performed in IQ-TREE v. 3.0.1 (Wong et al. 2025). Partitioning and substitution models were selected with the MFP+MERGE procedure in ModelFinder (Kalyaanamoorthy et al. 2017), starting from a scheme partitioned by marker. Node support was assessed with 1,000 SH-aLRT replicates (Guindon et al. 2010) and 10,000 ultrafast bootstrap replicates (Hoang et al. 2018) and is reported as SH-aLRT/UFBoot.

## Results

### Taxonomy

#### Ekueitermes

Taxon classificationAnimaliaIsopteraTermitidae

Duran-Bautista & Celis-Daza gen. nov.

A8B982AD-0977-5C1D-9BD0-72FDC5268FE6

https://zoobank.org/513C7A88-50F5-4233-B87D-5CA93A8B8D91

##### Type species.

*Ekueitermes alerosinus* sp. nov.

##### Diagnosis.

Mixed segment with a short, but not inflated, mesenteric tongue (Fig. 2C). P1 very short. The EVA of *Ekueitermes alerosinus* gen. et sp. nov. is composed of six conical cushions covered with 18–25 fringed polygonal scales, ranging from 4–15 each. The cushions are armed at the distal end with 7–17 sclerotised spines arranged in a palmate shape (Fig. 3A, B). The enteric valve (EV) has a well-differentiated, tubular enteric valve seating (EVS; Fig. 2E).

**Figure 1.**
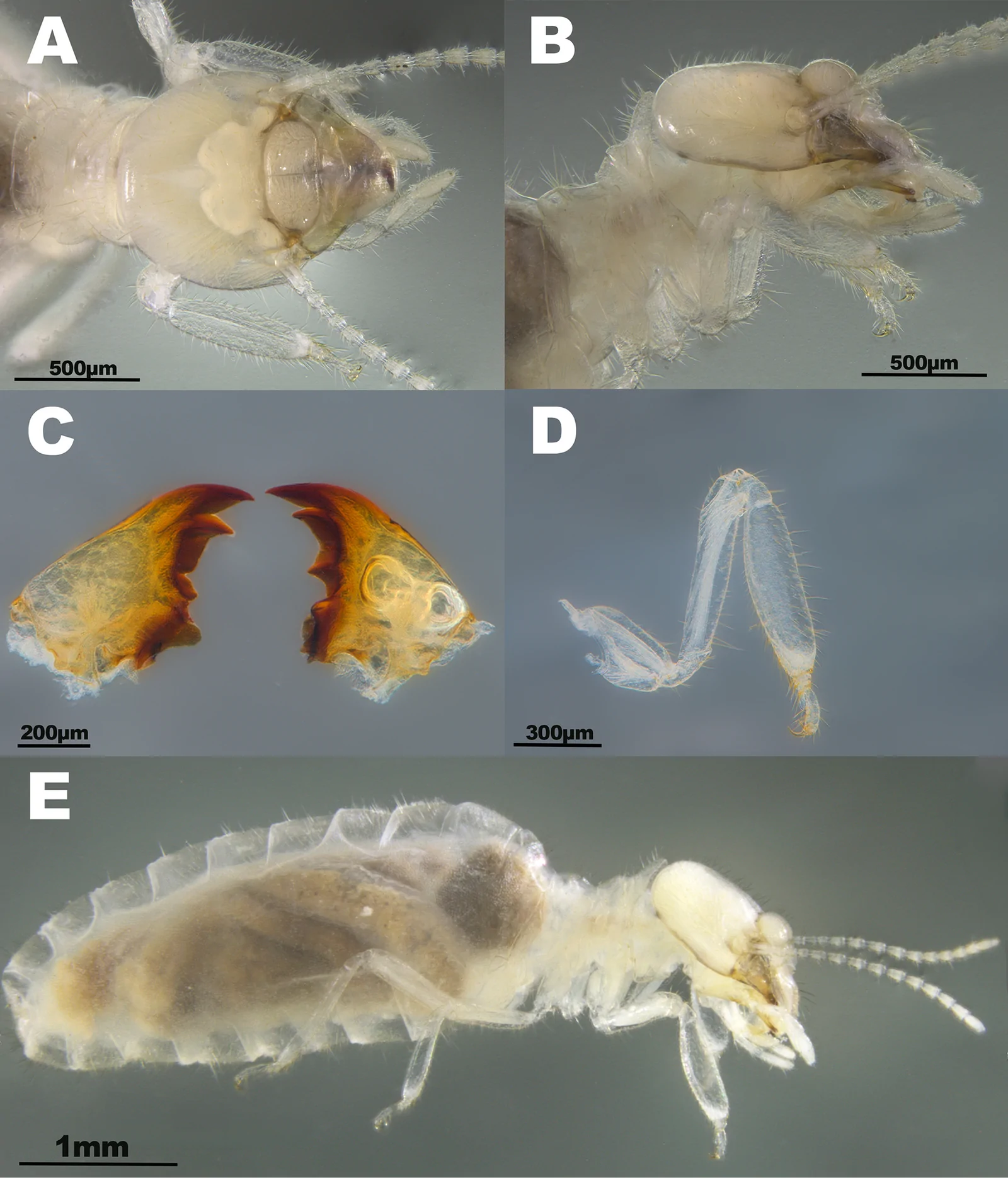
Worker of *Ekueitermes alerosinus* gen. et sp. nov. **A**. Dorsal view of the cephalic capsule; **B**. Lateral view of same; **C**. Dorsal view of the mandibles; **D**. Right anterior tibia; **E**. Right lateral habitus.

**Figure 2.**
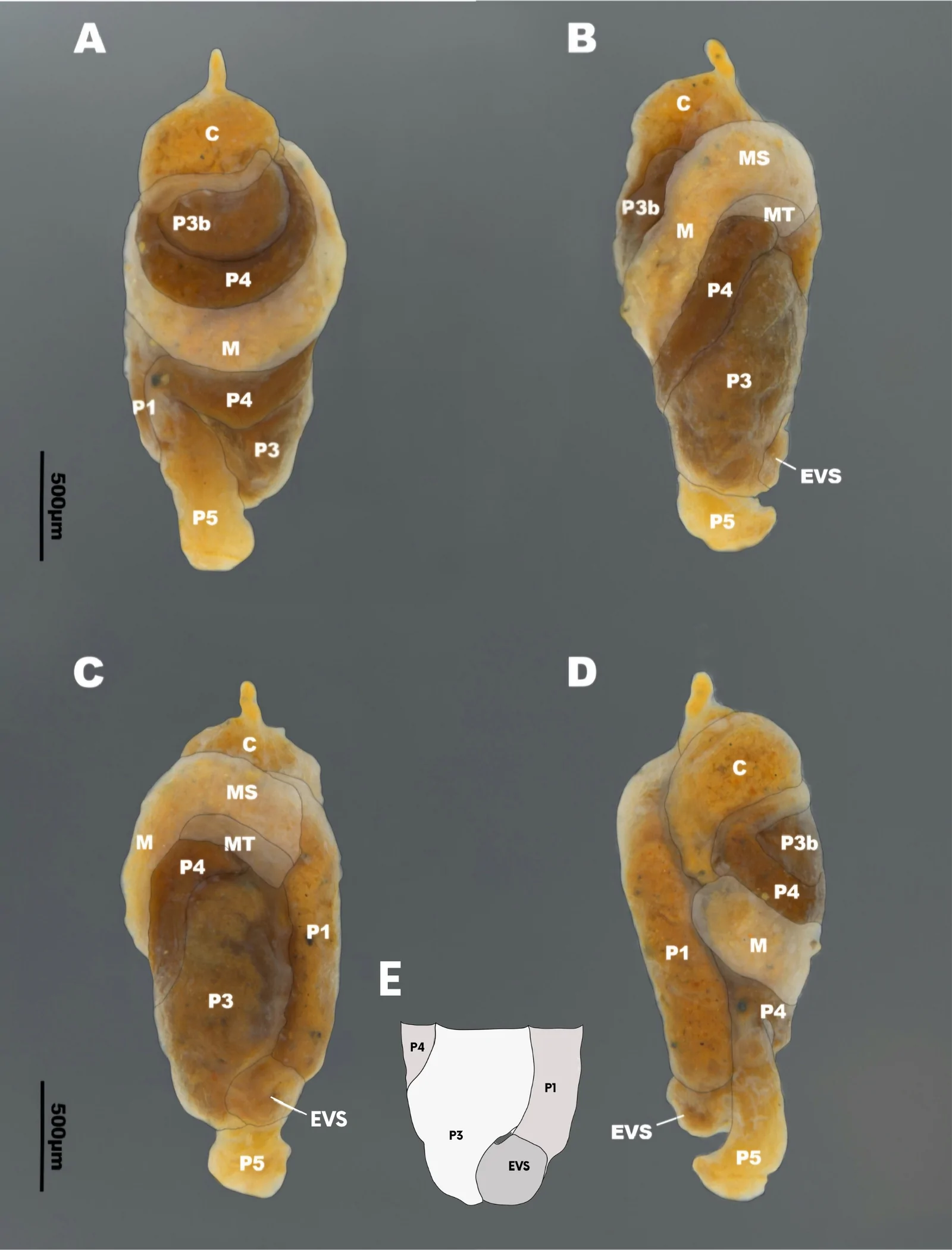
Digestive tract in a worker of *Ekueitermes alerosinus* gen. et sp. nov. **A**. Dorsal view; **B**. Right lateral view; **C**. Ventral view; **D**. Left lateral view; **E**. Diagrammatic detail of the enteric valve seating. Abbreviations: C = crop, M = mesenteron, MS = mixed segment, MT = mesenteric tongue, P1 = ileum, EVS = enteric valve seating, P3 and P3b = paunch, P4 = colon, P5 = rectum.

**Figure 3.**
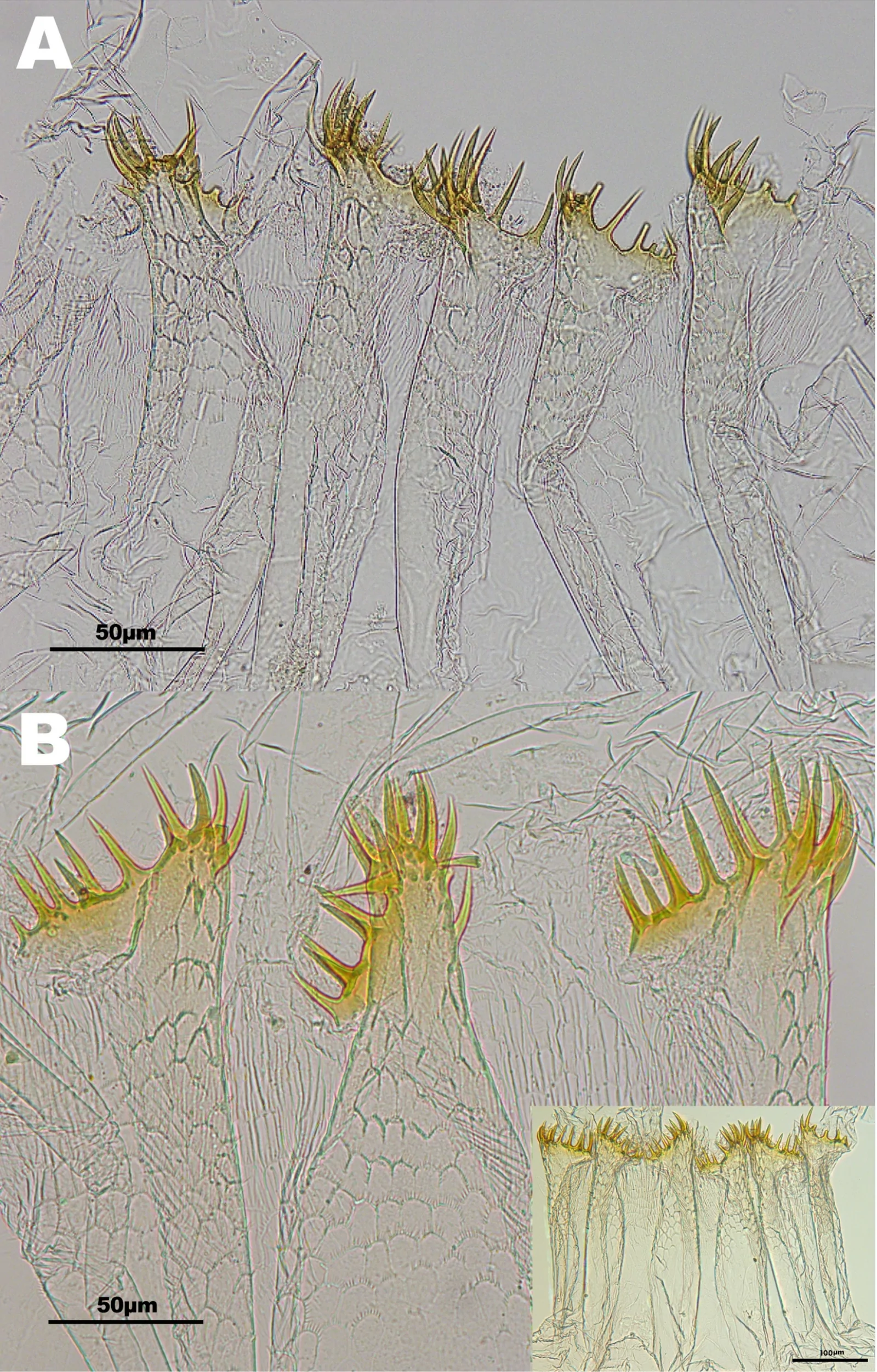
Enteric valve armature of *Ekueitermes alerosinus* gen. et sp. nov. (UFTC-CO869). **A**. Enteric valve fully stretched, showing the six cushions; **B**. Detail of cushions showing sclerotised spines arranged in a palmate shape, inset, general view of the enteric valve.

##### Description.

**Imago**. Unknown.

**Worker**. (Figs 1, 2, 3). Medium-sized (Fig. 1E), conspicuous and oval fontanelle, behind the brain (visible in preserved specimens) (Fig. 1A). Postclypeus strongly inflated; head pale white-yellowish with numerous uniformly spaced, long, fine, pale, erect setae interspersed with shorter setae (Fig. 1B). Antennae with 14 articles (formula 2>3<4<5). Left mandible with apical tooth prominent compared to M1+2; gap between the two forming an acute angle, posterior margin of M1+2 weakly concave (Fig. 1C); M3 (third marginal) less prominent than M1+2, edges forming an acute angle; well-developed molar prominence, not concealing molar process in dorsal view. Right mandible with apical tooth prominent compared to M1; small M1, slightly exceeding M2; gap between M1 and M2 forming right angle, M2 with concave posterior margin; without ridges. Pronotum saddle-shaped; anterior and posterior margins with a fringe of long, fine, erect setae. Anterior lobe broader than long, and posterior lobe is short and broad; meso-and metanotum also with fringes of setae on anterior margins. Middle fore-tibiae strongly inflated and hind legs slender with numerous long, pale, fine, erect setae on coxae, femora, tibiae, and tarsi (Fig. 1D), with a few long setae interspersed with numerous minute, densely packed setae; tibial spines weakly developed on all legs and not evident except distally; tibial spur formula 2:2:2.

Digestive tube with voluminous crop (Fig. 2D); mixed segment with a short, but not inflated, mesenteric tongue (Fig. 2B, C); midgut short and slender, widening into a very short and tubular P1, not forming a ventral loop; EVS well differentiated and tubular (Fig. 2E); EV armed, composed of six elongated, lightly sclerotised cushions, each terminating distally in a cluster of 7–17 straight to slightly curved, strongly sclerotised spines arranged in a palmate shape (Fig. 3A). Cushions distally narrow and then abruptly expanded at the point where the spines arise, cushion surface covered by ~50 polygonal, scale-like cells, each bearing a distal fringe of fine, short filaments (Fig. 3B). Intercushion area composed basally of fringed squamous cells and anteriorly of narrow, longitudinally striated cells. P3 large and voluminous; P4 very long and slender (Fig. 2A), abruptly broadening into a sac-like P5 (Fig. 2D).

##### Comparison and remarks.

The digestive tube morphology of *Ekueitermes* gen. nov. resembles that of *Patawatermes* Bourguignon & Roisin, 2016 and *Hirsutitermes* Scheffrahn, Carrijo & Castro, 2023. However, *Ekueitermes* gen. nov. possesses a shorter EVS, whereas in *Patawatermes*, the EVS is a prominent, externally visible tubular extension. Furthermore, *Hirsutitermes* exhibits a very long EVS characterised by three small, distinctive lobes, which are absent in *Ekueitermes* gen. nov.

While the EV of *Ekueitermes* gen. nov. may resemble that of *Patawatermes*, they are easily distinguished because *Patawatermes* cushions bear approximately 50–80 scales with posterior spiny fringes, whereas *Ekueitermes* gen. nov. presents only 18–25 fringed polygonal scales. Additionally, the EVA of *Patawatermes* consists of six rounded spiny cushions, unlike *Ekueitermes* gen. nov., where each cushion is armed at the distal end with 7–17 long, sclerotised spines in a palmate shape.

Although *Disjunctitermes* Scheffrahn, 2017 was recovered as the sister group of *Ekueitermes* gen. nov. (Fig. 5), the two genera are readily distinguished by their gut morphology. *Disjunctitermes* has a spheroidal mesenteric tongue and a large, robustly trilobed EVS, whereas *Ekueitermes* gen. nov. has a short, non-inflated mesenteric tongue and a well-differentiated tubular EVS. In addition, the EV of *Disjunctitermes* lacks sclerotised, spiny armature altogether, whereas in *Ekueitermes* gen. nov. each cushion terminates in a palmate cluster of 7–17 strongly sclerotised spines.

**Figure 4.**
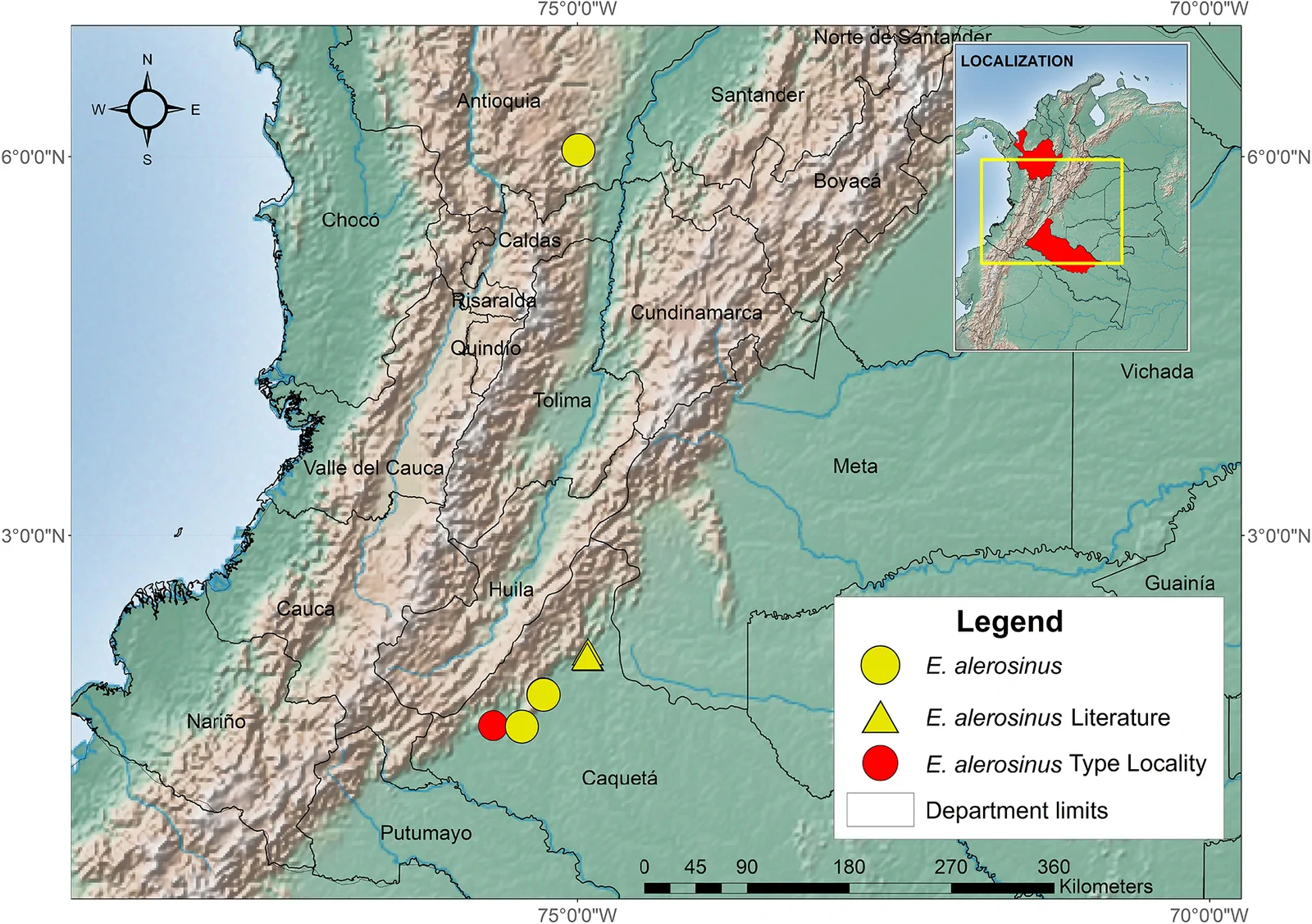
Distribution map of *Ekueitermes alerosinus* gen. et sp. nov. from literature data, UFTC, and LEUA records. The red circle indicates the type locality, and the yellow symbols represent additional records. In the localisation map inset, the red shaded areas represent the departments of Antioquia in the north and Caquetá in the south.

**Figure 5.**
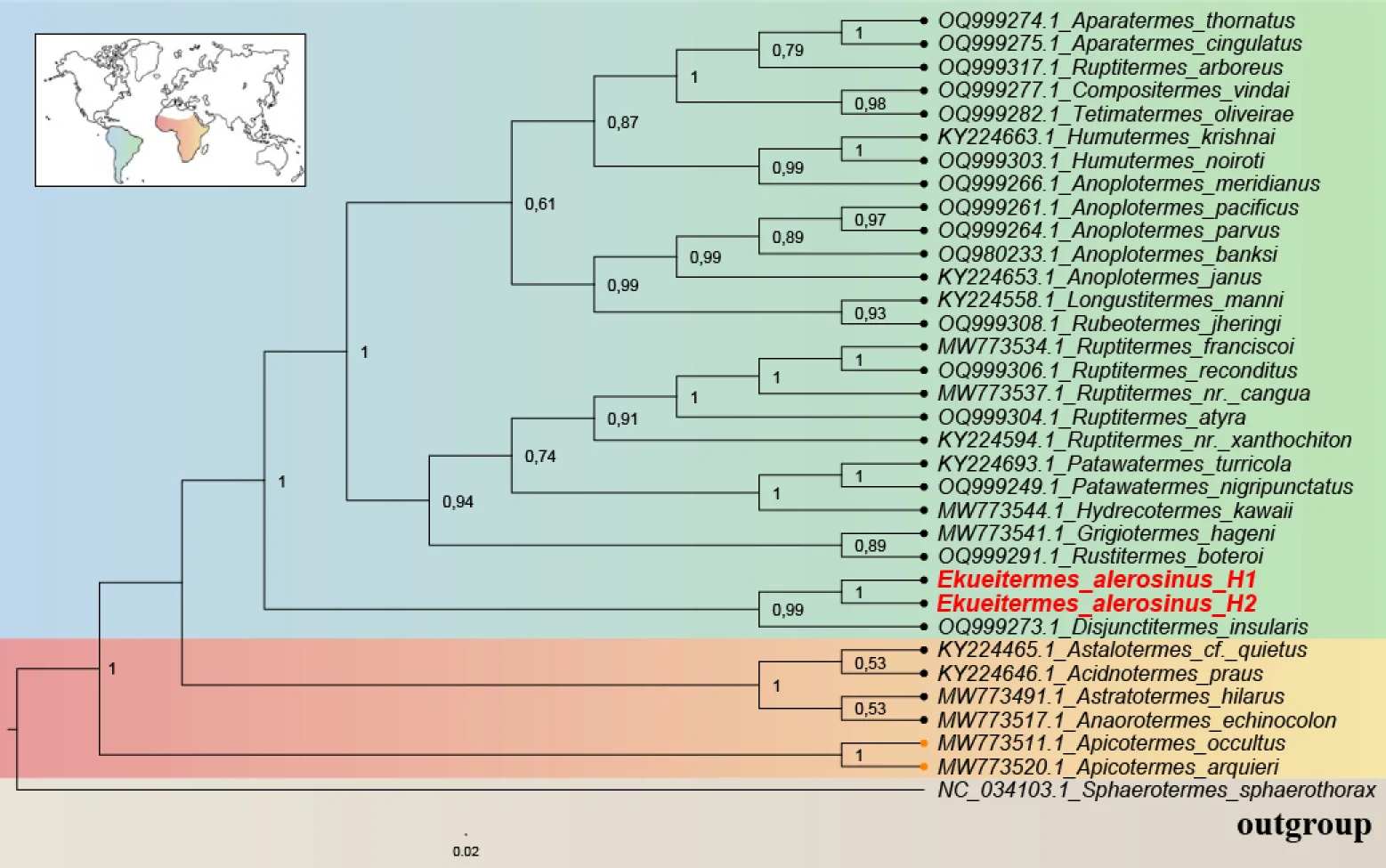
Bayesian inference tree of the subfamily Apicotermitinae based on mitochondrial COI and 16S genes. The analysis was performed in BEAST v. 2.5 under a Yule speciation prior and the TN93 substitution model. Values on branches indicate Bayesian posterior probabilities. Orange dots indicate species with soldiers, black dots soldierless species. *Sphaerotermes sphaerothorax* was used as the outgroup.

##### Molecular analysis.

The haplotypes obtained (H1 and H2) formed a strongly supported monophyletic clade (SH-aLRT / UFBoot = 100 / 100). This clade appears as a sister group to *Disjunctitermes insularis* Scheffrahn, 2017, a relationship that is also recovered under both criteria (PP = 0.99; 77/92).

##### Etymology.

From the Uitoto word *eeko* (phonetically *eekuei*), meaning spine, refers to the spines located on the distal portion of the EV cushions in workers and honours the Uitoto indigenous community inhabiting the Colombian Amazon.

#### Ekueitermes alerosinus

Taxon classificationAnimaliaIsopteraTermitidae

Duran-Bautista & Celis-Daza sp. nov.

BBBD60CE-FC1E-5B74-90B3-F3E261FB5ADE

https://zoobank.org/BE3E3738-B02D-4796-966B-D5F4823AFD5B

##### Material examined.

***Holotype***. • Worker (LEUA-22581) in a separate vial with the rest of the sample.

##### Type locality.

Colombia. Caquetá. Florencia. La Viciosa. Centro de Investigaciones Amazónicas Cimaz. Macagual, 1.4969, −75.6661.

##### Type repository.

University of the Amazon, Central Taxonomic Collection in liquid at the “Laboratorio de Entomología de la Universidad de la Amazonia” (LEUA).

##### Paratypes.

Colombia—Caquetá • Florencia, Village La viciosa, farm Centro de Investigaciones Amazónicas Cimaz. Macagual (1.4969, −75.6661), 17 Feb. 2017, 269 m, E. Durán coll.; 5 workers (LEUA-22581) • Puerto Rico, Village La Tigra, farm La Cabaña (1.7894, −75.2025), 08 Aug. 2018, 310 m, E. Durán coll.; 4 workers (LEUA-22582) • Montañita, Village Victoria Alta, farm Pekín (1.4875, −75.4375), 23 Feb. 2018, 260 m, E. Durán coll.; 11 workers (LEUA-22585) • El Doncello, Village La Ceiba, farm El Volga (1.7425, −75.2686), 02 Sep. 2018, 342 m, E. Durán coll.; 13 workers (LEUA-22583) • El Doncello, Village La Ceiba, farm El Volga (1.7394, −75.2666), 02 Sep. 2018, 342 m, E. Durán coll.; 7 workers (LEUA-22584)—Antioquia • San Luis (6.0499, −74.9925), 21 Jan. 1996, 998 m, J. Krecek coll.; 6 workers (UFTC-CO869).

##### Diagnosis.

As described for the genus.

##### Description.

**Imago**. Unknown.

**Worker** (Figs 1, 2, 3; Table 1). Monomorphic. Medium-sized. Body and head size varies from medium-sized to large. Pale white-yellowish cephalic capsule, covered with numerous uniformly spaced, long, fine, pale, erect setae interspersed with shorter setae; slightly concave dorsal surface in lateral view. Antennal articles translucent white. Pronotum with a fringe of long, fine, erect setae along anterior and posterior margins; several long setae on lateral margins. Mesonotum and metanotum with fringes of setae distributed along the margin of the anterior lobe. Transparent abdomen, tergites, and sternites densely covered with bristles of varying lengths. Forecoxa and anterior femur with some with numerous pale, fine, erect setae of varying sizes. Fore-tibiae, strongly inflated with a few long setae interspersed with numerous minute, densely packed setae; tibial spines weakly developed on all legs and not evident except distally; tibial spur formula 2:2:2.

**Table 1.** Measurements (mm) of 3 colonies (*n* = 17) of *Ekueitermes alerosinus* gen. et sp. nov. L = length, W = width.

	**Length of head to lateral base of mandibles**			**Width of head**			**Fore tibia W**			**Fore tibia L**			**Ratio foretibia W/L**		
	**Range**	**Mean**	**SD**	**Range**	**Mean**	**SD**	**Range**	**Mean**	**SD**	**Range**	**Mean**	**SD**	**Range**	**Mean**	**SD**
Holotype		0.7			0.86			0.21			0.68			0.31	
LEUA-22583 (3)	0.72–0.74	0.73	0.011	0.86–0.9	0.88	0.02	0.19–0.21	0.20	0.01	0.66–0.68	0.67	0.011	0.28–0.31	0.30	0.015
LEUA-22584 (8)	0.74–0.76	0.75	0.010	0.86–0.9	0.88	0.016	0.19–0.21	0.19	0.007	0.66–0.68	0.67	0.010	0.28–0.31	0.29	0.011
UFTC-CO869 (6)	0.70–0.80	0.77	0.044	0.91–0.96	0.92	0.035	0.17–0.21	0.19	0.014	0.69–0.77	0.73	0.031	0.23–0.29	0.26	0.023

Digestive tube with a voluminous crop narrowing toward the gizzard, mixed segment (MS) with a short, but not inflated, mesenteric tongue. Width of the first proctodeal segment (P1) uniform, narrowing toward the apex of the EVS, EVS well-differentiated and tubular, separate from P3. Medium-sized and tubular EV with six elongated, lightly sclerotised cushions, each with a conical shape, wider at the base (near P1) and narrower at the apex (near P3), with ~50 fringed, polygonal scales ranging from 4 to 15 each. Each pad is armed at the distal end with 7–17 straight to slightly curved, strongly sclerotised spines arranged in a palmate shape. The proximal portion of the pad has a small spine directed distally (near P1) or, occasionally, one of the cushions may have up to two spines the intercushion area with 13–22 scales similar to those on the pads (Fig. 3B; Suppl. material 2).

##### Comparison and remarks.

See genus remarks above.

##### Etymology.

The specific epithet alerosinus is after Alejandro Duran, Leofrey Duran, and Rosario Bautista (son and parents of the first author), whose names are combined in the root *Alero*, in recognition of their unconditional support and affection throughout his training as a researcher.

##### Ecology and distribution.

The species was mainly collected in the soil, up to 20 cm deep. Like many Apicotermitinae in the New World, it does not build above-ground structures. It inhabits a variety of environments, including natural forests, pastures, and agroforestry systems. Geographically, the species is distributed from the Amazonian foothills in the department of Caquetá to the Central Andes in the department of Antioquia. Consequently, the species exhibits a broad altitudinal range, occurring from 260 m in the lowlands to 998 m above sea level in the Andean region (Fig. 4). In the Andean region, it has been recorded in association with *Dolichorhinotermes longidens*. Previous literature records for this species in Caquetá were reported by Castro et al. (2021) under the provisional name Apicotermitinae sp. 4, a designation consistent with the habitat data and paratypes examined in the present study.

##### Molecular analysis and phylogenetic relationships.

Sequencing yielded one COI haplotype (Suppl. material 3) and two 16S haplotypes (Suppl. material 4). Standard IUPAC ambiguity codes (Y, R, K) were consistently recovered at a few COI positions across all replicates and in both reading directions, representing intra-sample polymorphism expected from pooled individuals. The final concatenated matrix comprised 941 bp for 34 terminals.

BI recovered *Ekueitermes alerosinus* gen. et sp. nov. (H1, H2) as a monophyletic clade with maximum support (PP = 1), placed as sister to *Disjunctitermes insularis* (PP = 0.99; Fig. 5). Maximum likelihood recovered the same two relationships (100/100 and 77/92, respectively; Suppl. material 5). The two methods differed in the placement of *Grigiotermes hageni* (Snyder & Emerson, 1949), *Rustitermes boteroi* Castro et al., 2020, and *Anoplotermes parvus* Snyder, 1923. Deeper nodes within the Neotropical clade were weakly supported under both criteria.

Genetic distances (Suppl. material 6) were minimal between the two haplotypes obtained here (K2P = 0.0011), confirming that both belong to a single lineage. The closest relative was *D. insularis* (K2P = 0.1007–0.1019), a divergence exceeding that observed between several genera included in this analysis; 56 intergeneric pairs showed lower values, including *Hydrecotermes kawaii* and *Patawatermes nigripunctatus* (0.0702), *Patawatermes turricola* and *H. kawaii* (0.0790), and *Longustitermes manni* and *Anoplotermes parvus* (0.0838).

## Discussion

The description of *Ekueitermes alerosinus* gen. et sp. nov. highlights this gap, since, despite being found in relatively common habitats in the region, it had not been previously recognised. This suggests that the actual richness of Apicotermitinae in the Amazon biome remains underestimated, aligning with the findings of Bourguignon et al. (2016a, 2016b) and Castro et al. (2020), who highlighted a significant taxonomic gap in the Neotropical region.

When compared to other Apicotermitinae species widely distributed in South America, such as *Rustitermes boteroi*, it is evident that many taxa possess broader distribution ranges than initial records suggest, succumb to the Wallacean Shortfall (Lomolino 2004). Recent ecological studies and inventories have incorporated photographs of enteric valves of Apicotermitinae morphotypes (Dahlsjö et al. 2020; Ferreira et al. 2023, 2025), which have served as supporting evidence for reporting additional records in species descriptions (Carrijo et al. 2023). In the case of *E. alerosinus* gen. et sp. nov., the species has been reported only in a single study near the type locality (Castro et al. 2021); however, it is noteworthy that it was recorded in two different biogeographic regions (Amazonia and Andes), suggesting a wider distribution than previously reported, particularly given that the Andean region is one of the least sampled areas in the country (Pinzón et al. 2019). The characterisation of the EV was essential to differentiate this species from related genera, reaffirming the diagnostic importance of this character in the identification of soldierless termites (Bourguignon et al. 2013; Dahlsjö et al. 2020). However, limitations arising from photographic material or insufficiently detailed descriptions have in the past made it difficult to correctly delimit species (Castro et al. 2021), suggesting the importance of generating comprehensive descriptions accompanied by high-quality images.

The phylogenetic topology recovered is consistent with previous evolutionary frameworks proposed for Apicotermitinae (Scheffrahn et al. 2017; Castro et al. 2020; Romero Arias et al. 2021; Carrijo et al. 2023). Within this structure, the H1–H2 lineage forms a monophyletic clade with maximum support, positioned as the sister group to *Disjunctitermes insularis* Scheffrahn, 2017 under both inference criteria. Notably, the genetic distance between these two lineages exceeds the divergence observed between several established genera, such as *Longustitermes* Bourguignon & Roisin, 2010, *Anoplotermes* Müller, 1873, and *Patawatermes*. This high level of divergence, combined with a phylogenetic position consistently recovered by both methods and the diagnostic characters of the digestive tube described above, identifies the H1–H2 lineage as an independent evolutionary unit, supporting the erection of *Ekueitermes alerosinus* gen. et sp. nov.

The topological differences observed between the two inference criteria involved genera whose placement has proved unstable in previous analyses. *Grigiotermes* has been recovered in different positions depending on the markers and inference method employed (Scheffrahn et al. 2017; Castro et al. 2020; Romero Arias et al. 2021; Carrijo et al. 2023). By contrast, the position of *Ekueitermes* gen. nov. was identical under both criteria, and its sister-group relationship with *Disjunctitermes* was recovered regardless of the optimality criterion applied.

Despite being recovered as sister taxa, *Ekueitermes* gen. nov. and *Disjunctitermes* differ markedly in their EVA. That of *Ekueitermes* gen. nov. is more like those of *Patawatermes* and *Hirsutitermes*, whereas *Disjunctitermes* lacks sclerotised armature altogether. A comparable mismatch was reported by Carrijo et al. (2023), who found that the EVA and COI alone suggested a new genus for *Anoplotermes susanae*, whereas gut morphology and the complete mitogenome confirmed its placement within *Anoplotermes*. This adds to evidence that EVA alone may be insufficient to infer relationships within the subfamily and supports the combined use of additional gut characters and molecular data.

However, resolving the evolution of Neotropical soldierless lineages remains a challenge. While COI provided a reliable taxonomic proxy, single mitochondrial markers can be insufficient to resolve deeper relationships, as evidenced by the paraphyly detected in genera like *Anoplotermes* and *Astalotermes* Sands, 1972 (Scheffrahn et al. 2017). In the present study, COI and 16S rRNA were combined to increase the number of informative positions, yet deeper nodes within the Neotropical clade remained weakly supported under both criteria. Nevertheless, future mitogenomic or phylogenomic analyses will be essential to confirm if the current placement of *Ekueitermes* gen. nov. remains stable or if broader genomic sampling reveals deeper affinities with other lineages.

The formal description of *Ekueitermes* gen. nov. addresses a significant information gap in the taxonomic and ecological records of Neotropical Apicotermitinae. As previously noted in regional surveys where this lineage remained unnamed, formal taxonomic identification is necessary to document biodiversity in regions undergoing land-use changes, such as the Amazonian foothills of Caquetá and the Colombian Andes.

## Supplementary Material

XML Treatment for Ekueitermes

XML Treatment for Ekueitermes alerosinus

## Additional information

### Artificial Intelligence (AI) use

The authors accept full responsibility for the content of the manuscript, including the disclosure of any use of AI.

No AI tools were used in the preparation of this manuscript.

### Author ORCIDs

E.H. Duran-Bautista https://orcid.org/0000-0002-4155-6350

F. Celis-Daza https://orcid.org/0009-0005-9354-4406

N.S. Poveda Quezada https://orcid.org/0009-0001-1625-3163

F.A. Ortiz-Morea https://orcid.org/0000-0003-0978-1256

R.H. Scheffrahn https://orcid.org/0000-0002-6191-5963

D. Castro https://orcid.org/0000-0002-5060-6129

### Data availability

All of the data that support the findings of this study are available in the main text or Supplementary Information.

## Supplementary materials

Supplementary material 1 List of GenBank accession numbers Data type docx Explanation note **table SS1**. List of GenBank accession numbers for complete mitochondrial genomes of the subfamily Apicotermitinae and outgroup taxa included in the phylogenetic analyses. https://binary.pensoft.net/file/1778880This dataset is made available under the Open Database License (http://opendatacommons.org/licenses/odbl/1.0/). The Open Database License (ODbL) is a license agreement intended to allow users to freely share, modify, and use this Dataset while maintaining this same freedom for others, provided that the original source and author(s) are credited. Ervin H. Duran-Bautista, Fernando Celis-Daza, Nasly S. Poveda Quezada, Fausto A. Ortiz-Morea, Rudolf H. Scheffrahn, Daniel Castro

Supplementary material 2 Enteric valve of workers Data type png Explanation note **fig. S1**. Enteric valve of workers *Ekueitermes alerosinus* gen. et sp. nov. (LEUA-22584). **A**. The red circle highlights a small spine that is occasionally present in the proximal portion of the cushions (near P1); **B**. Detail of the cushions. https://binary.pensoft.net/file/1778881This dataset is made available under the Open Database License (http://opendatacommons.org/licenses/odbl/1.0/). The Open Database License (ODbL) is a license agreement intended to allow users to freely share, modify, and use this Dataset while maintaining this same freedom for others, provided that the original source and author(s) are credited. Ervin H. Duran-Bautista, Fernando Celis-Daza, Nasly S. Poveda Quezada, Fausto A. Ortiz-Morea, Rudolf H. Scheffrahn, Daniel Castro

Supplementary material 3 Alignment of the COI region Data type png Explanation note **fig. S2**. Alignment of the COI region. Red boxes highlight ambiguous nucleotide positions coded according to the IUPAC ambiguity system: Y (C/T), R (A/G), and K (G/T). https://binary.pensoft.net/file/1778882This dataset is made available under the Open Database License (http://opendatacommons.org/licenses/odbl/1.0/). The Open Database License (ODbL) is a license agreement intended to allow users to freely share, modify, and use this Dataset while maintaining this same freedom for others, provided that the original source and author(s) are credited. Ervin H. Duran-Bautista, Fernando Celis-Daza, Nasly S. Poveda Quezada, Fausto A. Ortiz-Morea, Rudolf H. Scheffrahn, Daniel Castro

Supplementary material 4 Alignment of the 16S region Data type png Explanation note **fig. S3**. Alignment of the 16S region. Sequences with constant length in all taxa. https://binary.pensoft.net/file/1778883This dataset is made available under the Open Database License (http://opendatacommons.org/licenses/odbl/1.0/). The Open Database License (ODbL) is a license agreement intended to allow users to freely share, modify, and use this Dataset while maintaining this same freedom for others, provided that the original source and author(s) are credited. Ervin H. Duran-Bautista, Fernando Celis-Daza, Nasly S. Poveda Quezada, Fausto A. Ortiz-Morea, Rudolf H. Scheffrahn, Daniel Castro

Supplementary material 5 Maximum-likelihood tree of the subfamily Apicotermitinae Data type png Explanation note **fig. S4**. Maximum-likelihood tree of the subfamily Apicotermitinae based on the mitochondrial COI and 16S genes. Partitioning and substitution models were selected with the MFP+MERGE procedure in ModelFinder. Support values on branches represent SH-aLRT (1,000 replicates) and ultrafast bootstrap (10,000 replicates), respectively. Orange dots indicate species with soldiers; black dots indicate soldierless species. *Sphaerotermes sphaerothorax* was used as the outgroup. https://binary.pensoft.net/file/1778884This dataset is made available under the Open Database License (http://opendatacommons.org/licenses/odbl/1.0/). The Open Database License (ODbL) is a license agreement intended to allow users to freely share, modify, and use this Dataset while maintaining this same freedom for others, provided that the original source and author(s) are credited. Ervin H. Duran-Bautista, Fernando Celis-Daza, Nasly S. Poveda Quezada, Fausto A. Ortiz-Morea, Rudolf H. Scheffrahn, Daniel Castro

Supplementary material 6 Pairwise K2P genetic distances Data type xlsx Explanation note **table S2**. Pairwise K2P genetic distances among the 34 terminals included in the phylogenetic analyses. https://binary.pensoft.net/file/1778885This dataset is made available under the Open Database License (http://opendatacommons.org/licenses/odbl/1.0/). The Open Database License (ODbL) is a license agreement intended to allow users to freely share, modify, and use this Dataset while maintaining this same freedom for others, provided that the original source and author(s) are credited. Ervin H. Duran-Bautista, Fernando Celis-Daza, Nasly S. Poveda Quezada, Fausto A. Ortiz-Morea, Rudolf H. Scheffrahn, Daniel Castro
