# Supplementary material for: Ekueitermes alerosinus, a new genus and species of soldierless termite from Colombia (Isoptera, Termitidae, Apicotermitinae)
Source: Zookeys. 2026 Sep 18;1292:275–92. doi: 10.3897/zookeys.1292.195853 (PMC13615400; doi:10.3897/zookeys.1292.195853)
Supplement: Supplementary file 1 [file zookeys-1292-275_article-195853__-s001.docx]

**Supplementary material**

**Table S1.** List of GenBank accession numbers for complete mitochondrial genomes of the subfamily Apicotermitinae and outgroup taxa included in the phylogenetic analyses.

| **Especie** | **Codigo GenBank** | **Referencia** | **DOI** |
| --- | --- | --- | --- |
| *Acidnotermes praus* | KY224646.1 | Romero et al., 2021 | https://doi.org/10.1111/syen.12486 |
| *Aderitotermes_cavator* | MW773504 | Romero et al., 2021 | https://doi.org/10.1111/syen.12486 |
| *Alyscotermes_kilimandjaricus* | NC_034022.1 | Bourguignon et al., 2017 | <https://doi.org/10.1093/molbev/msw253> |
| *Amalotermes_phaeocephalus* | NC_034099 | Bourguignon et al., 2017 | <https://doi.org/10.1093/molbev/msw253> |
| *Anaorotermes_echinocolon* | MW773517 | Romero et al., 2021 | https://doi.org/10.1111/syen.12486 |
| *Anenteotermes nanus* | MW773495 | Romero et al., 2021 | https://doi.org/10.1111/syen.12486 |
| *Anenteotermes polyscolus* | MW773514 | Romero et al., 2021 | https://doi.org/10.1111/syen.12486 |
| *Anoplotermes_banksi* | OQ980233.1 | Arora et al., 2023 | <https://doi.org/10.1098/rspb.2023.0619> |
| *Anoplotermes_meridianus* | OQ999266.1 | Carrijo et al., 2023 | https://doi.org/10.3897/zookeys.1167.100001 |
| *Anoplotermes_pacificus* | OQ999262.1 | Carrijo et al., 2023 | https://doi.org/10.3897/zookeys.1167.100001 |
| *Anoplotermes parvus* | KY224649.1 | Romero et al., 2021 | https://doi.org/10.1111/syen.12486 |
| *Aparatermes_cingulatus* | OQ999276.1 | Carrijo et al., 2023 | https://doi.org/10.3897/zookeys.1167.100001 |
| *Apicotermes arquieri* | MW773520 | Romero et al., 2021 | https://doi.org/10.1111/syen.12486 |
| *Apicotermes occultus* | MW773511 | Romero et al., 2021 | https://doi.org/10.1111/syen.12486 |
| *Apolemotermes fodiens* | OQ999303.1 | Romero et al., 2021 | https://doi.org/10.1111/syen.12486 |
| *Asagarotermes coronatus* | MW773530 | Romero et al., 2021 | https://doi.org/10.1111/syen.12486 |
| *Astalotermes cf. brevior* | MW773521 | Romero et al., 2021 | https://doi.org/10.1111/syen.12486 |
| *Astalotermes murcus* | KY224676.1 | Romero et al., 2021 | https://doi.org/10.1111/syen.12486 |
| *Astalotermes quietus* | KY224465.1 | Romero et al., 2021 | https://doi.org/10.1111/syen.12486 |
| *Astratotermes hilarus* | MW773491 | Romero et al., 2021 | https://doi.org/10.1111/syen.12486 |
| *Compositermes vindai* | KY224716.1 | Romero et al., 2021 | https://doi.org/10.1111/syen.12486 |
| *Disjunctitermes insulares* | OQ999273.1 | Castro et al., 2023 | https://doi.org/10.3897/zookeys.1167.100001 |
| *Grigiotermes hageni* | MW773541 | Romero et al., 2021 | https://doi.org/10.1111/syen.12486 |
| *Humutermes krishnai* | KY224663.1 | Romero et al., 2021 | https://doi.org/10.1111/syen.12486 |
| *Humutermes noiroti* | OQ999303.1 | Castro et al., 2023 | https://doi.org/10.3897/zookeys.1167.100001 |
| *Hydrecotermes kawaii* | MW773544 | Romero et al., 2021 | https://doi.org/10.1111/syen.12486 |
| *Longustitermes manni* | KY224558.1 | Romero et al., 2021 | https://doi.org/10.1111/syen.12486 |
| *Patawatermes nigripunctatus* | KY224420.1 | Romero et al., 2021 | https://doi.org/10.1111/syen.12486 |
| *Patawatermes turricola* | KY224693.1 | Romero et al., 2021 | https://doi.org/10.1111/syen.12486 |
| *Ruptitermes atyra* | OQ999304.1 | Carrijo et al., 2023 | https://doi.org/10.3897/zookeys.1167.100001 |
| *Ruptitermes franciscoi* | MW773534 | Romero et al., 2021 | https://doi.org/10.1111/syen.12486 |
| *Ruptitermes nr. cangua* | MW773537 | Romero et al., 2021 | https://doi.org/10.1111/syen.12486 |
| *Ruptitermes reconditus* | OQ999305.1 | Castro et al., 2023 | https://doi.org/10.3897/zookeys.1167.100001 |
| *Rustitermes boteroi* | OQ999291.1 | Castro et al., 2023 | https://doi.org/10.3897/zookeys.1167.100001 |
| *Tetimatermes oliveirae* | OQ999282.1 | Castro et al., 2023 | https://doi.org/10.3897/zookeys.1167.100001 |
| *Sphaerotermes sphaerothorax* | NC_034103 | Bourguignon et al., 2017 | <https://doi.org/10.1093/molbev/msw253> |
